# A Case Report of Intradiaphragmatic Abscess

**DOI:** 10.70352/scrj.cr.25-0095

**Published:** 2025-07-03

**Authors:** Yuuki Matsui, Koji Takami, Reishi Toshiyama, Haruka Todoroki, Shinji Futami, Seigo Minami

**Affiliations:** 1 Department of General Thoracic Surgery, NHO Osaka National Hospital, Osaka, Osaka, Japan; 2 Department of Surgery, NHO Osaka National Hospital, Osaka, Osaka, Japan; 3 Department of Respiratory Medicine, NHO Osaka National Hospital, Osaka, Osaka, Japan

**Keywords:** intradiaphragm, abscess, laparoscopy, thoracoscopy, rare case

## Abstract

**INTRODUCTION:**

Intradiaphragmatic abscesses are extremely rare; therefore, making a correct preoperative diagnosis is difficult. Furthermore, their pathogenesis is not well understood because of the limited number of reported cases.

**CASE PRESENTATION:**

A 62-year-old Japanese male who had undergone cholecystectomy for acute cholecystitis complicated by cholelithiasis 1 year previously presented to our hospital with a fever and right chest pain. Laboratory investigations revealed an elevated inflammatory response. Preoperative computed tomography suggested an intra-abdominal abscess and right pyothorax, and surgical drainage was performed via laparoscopic and thoracoscopic approaches because there was no laboratory improvement after intravenous antibiotic therapy. Intraoperative findings showed a localized bulge in the right diaphragmatic dome without an abscess in the liver or the subdiaphragmatic area. A whitish pus was drained through an incision. By contrast, in the thoracic cavity, serous pleural effusion, fibrin precipitation, and localized bulge on the same diaphragmatic site as the abdominal bulge were found without abscess formation. Pus was not drained by puncture aspiration, and no incision was made. The pus culture was positive for *Escherichia coli*. A combined abdominal and thoracic approach allows for correct diagnosis and appropriate treatment. The patient’s general condition improved postoperatively, and he remained well without evidence of recurrence of the intradiaphragmatic abscess 18 months later for follow-up chest computed tomography.

**CONCLUSIONS:**

Despite the extremely rare nature of the disease, if an intradiaphragmatic abscess is suspected preoperatively, a combined abdominal and thoracic approach may be useful for making the correct diagnosis and carrying out appropriate treatment.

## Abbreviation

HDA
: high-density area

## INTRODUCTION

Intradiaphragmatic abscesses are extremely rare, and their pathogenesis is not well known. Until November 2024, five cases of intradiaphragmatic abscesses were reported in the English and Japanese literature.^1–5)^

We herein report a case of intradiaphragmatic abscess that was treated with incision and drainage. A combined abdominal and thoracic approach revealed a localized abscess within the diaphragm.

## CASE PRESENTATION

**Fig. 1:**
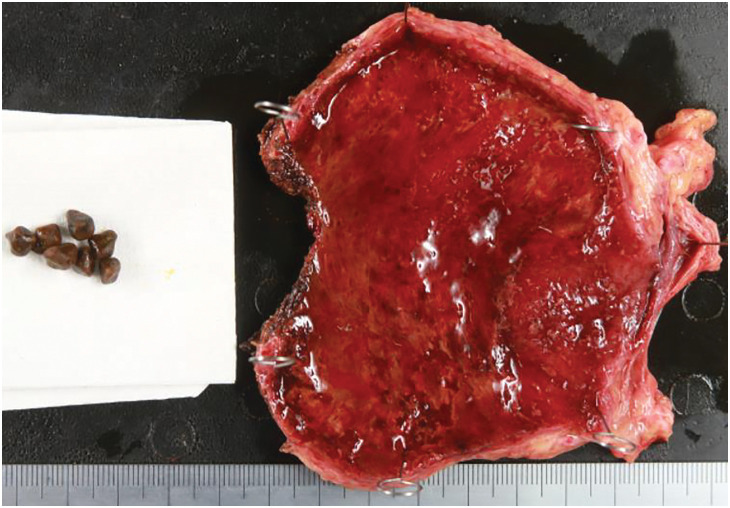
Macroscopic finding. Macroscopic findings post-cholecystectomy showed some gallstones.

**Fig. 2:**
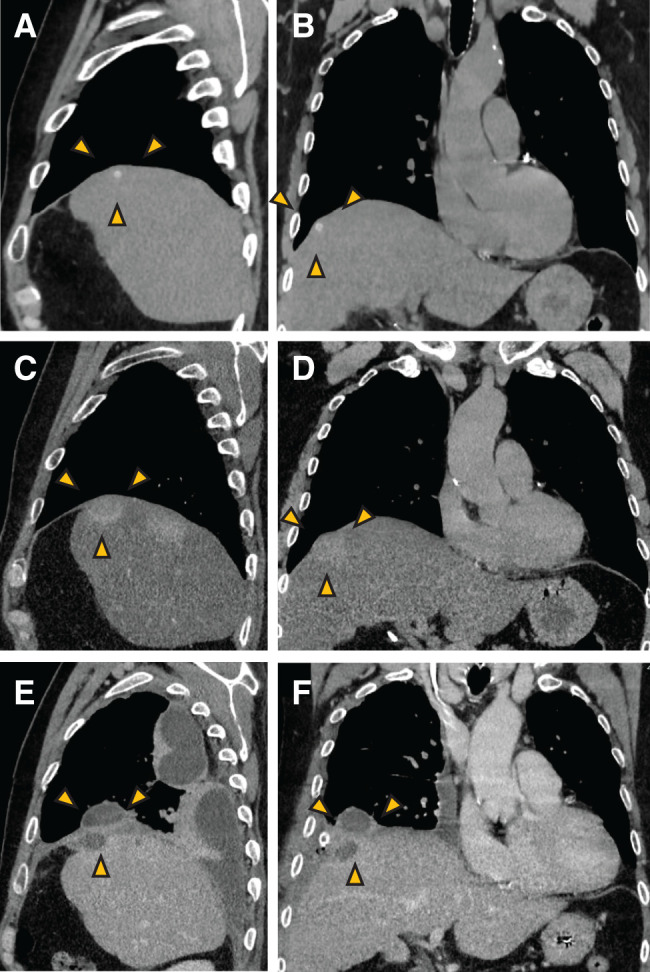
Course of preoperative CT images. (**A**, **B**) CT images 1 month after cholecystectomy. The images showed residual gallstone on the liver surface. The residual gallstone is indicated by yellow arrowheads. (**C**, **D**) CT images 6 months after cholecystectomy showing a HDA in the liver. The HDA is indicated by yellow arrowheads. (**E**, **F**) CT images 1 year after cholecystectomy. The images showed a right subdiaphragmatic fluid space suspicious of an intra-abdominal abscess and a pleural effusion suspicious of a right pyothorax. These areas are indicated by yellow arrowheads. HDA, high-density area

**Fig. 3:**
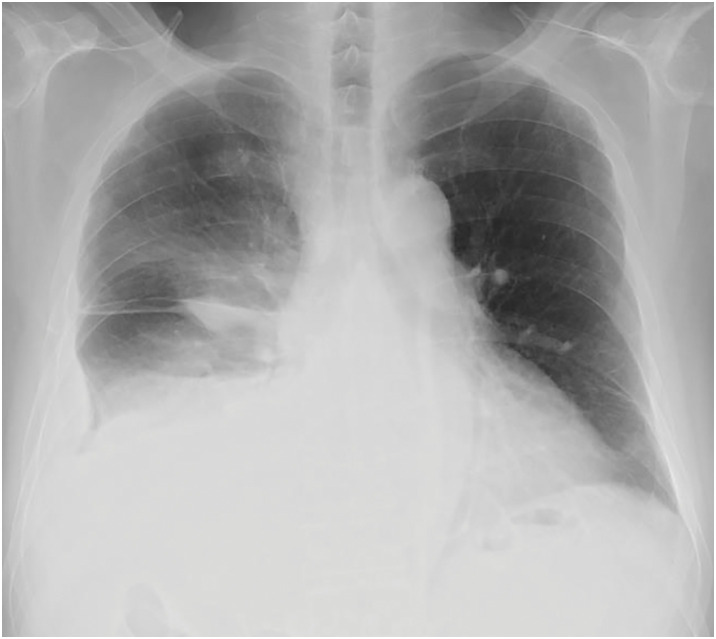
Chest X-ray image. Right hemidiaphragm elevation with right lower lobe atelectasis. Left hemithorax is normal.

A 62-year-old Japanese male presented to our hospital with a fever (40°C) and right-sided chest pain. He had undergone cholecystectomy for acute cholecystitis complicated by cholelithiasis 1 year previously (**Fig. 1**). CT, 1 month after surgery, showed residual gallstones in the subdiaphragmatic area (**Fig. 2A**, **2B**). Furthermore, CT performed 6 months after surgery showed an intrahepatic HDA in the same area where the residual gallstone was found (**Fig. 2C**, **2D**), and hepatic inflammatory pseudotumor was suspected; however, because there were no symptoms or abnormal blood tests, the patient was not followed up. At an emergency visit 1 year after surgery, laboratory investigation revealed a white cell count of 16.9 × 10^9^/L, neutrophils of 16.7 × 10^9^/L, platelets of 288 × 10^9^/L, hemoglobin of 15.0 g/dL, and C-reactive protein 30.4 mg/dL. Chest radiography revealed a right pleural effusion and an elevated right hemidiaphragm with atelectasis in the right lower lobe. The left lung was clearly visible (**Fig. 3**). Chest and abdominal CT showed a right subdiaphragmatic fluid space suspected of being an intra-abdominal abscess and pleural effusion suspected of being right pyothorax due to inflammation spillover (**Fig. 2E**, **2F**). Culture of both the blood and pleural effusions showed no growth of the organisms. Surgical drainage was deemed necessary because of the lack of improvement in laboratory findings after intravenous antibiotic therapy. Abdominal and thoracic surgeries were also performed.

**Fig. 4:**
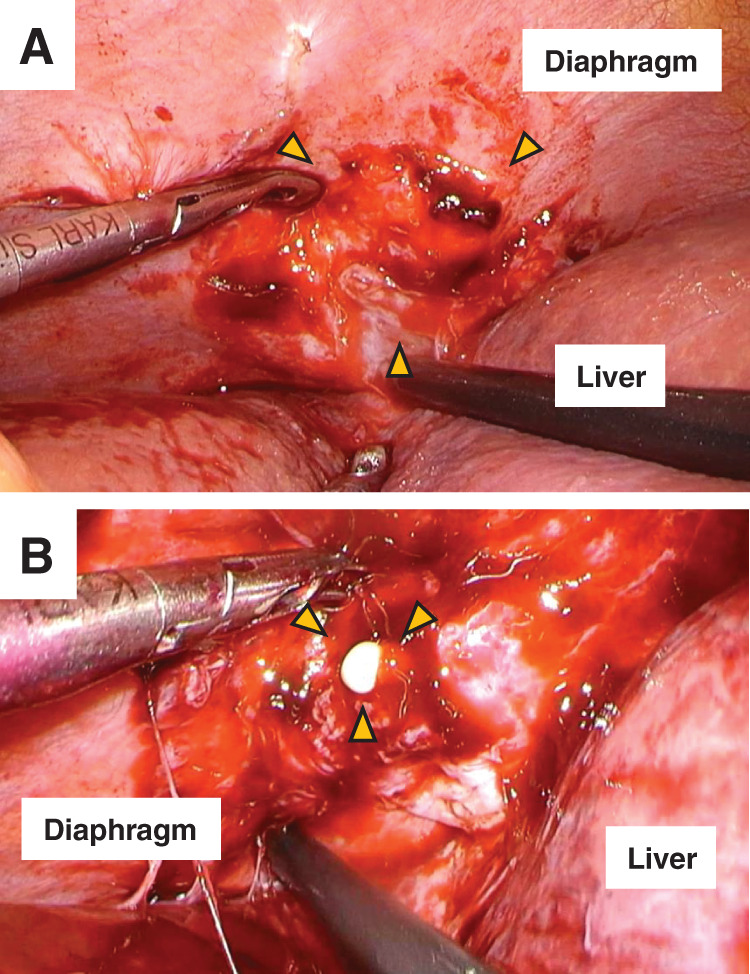
Intraoperative findings. (**A**) A localized bulge in the right diaphragmatic dome. (**B**) Drainage of whitish pus by incision. These areas are indicated by yellow arrowheads.

**Fig. 5:**
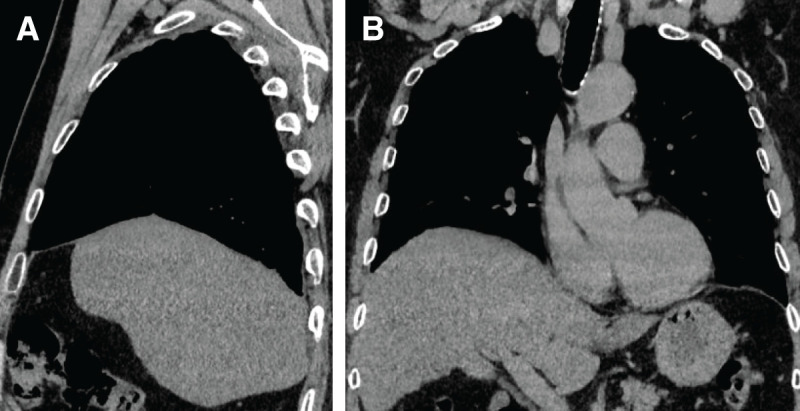
Postoperative CT images. (**A**, **B**) CT images 18 months after surgery. The images show that it remains well without evidence of recurrence of intradiaphragmatic abscess.

We confirmed the diagnosis of an intra-abdominal abscess and right pyothorax and performed thoracic and abdominal surgery. Abdominal surgery was followed by thoracic surgery, and the patient's position was changed from the supine to the left lateral decubitus position. No abscess was found in the liver or subdiaphragmatic area. We found a 4 × 4-cm localized bulge in the right diaphragmatic dome (**Fig. 4A**). Whitish pus amounting to 5 mL was drained through an incision in the diaphragm (**Fig. 4B**). After pus drainage, the intra-abdominal space was irrigated with saline solution. Right thoracoscopic surgery revealed only serous pleural effusion and fibrin precipitation without abscess formation in the thoracic cavity. Although a localized bulge was also observed at the same site of the diaphragm as the abdominal bulge, no pus was drained by puncture aspiration; therefore, no incision was made. The pus culture was positive for *Escherichia coli*. One day after the surgery, his temperature returned to normal. The amount of fluid in the chest drainage tube is minimal. The two drainage tubes placed between the liver and the incision of the diaphragm and in the thoracic space were subsequently removed and remained well without diaphragm dysfunction or evidence of recurrence of intradiaphragmatic abscess 18 months later on follow-up chest CT (**Fig. 5**).

## DISCUSSION

There have been only a few reports of intradiaphragmatic abscesses,^1–5)^ three of which were published before 1990.^1–3)^ This report is valuable because the case described here is extremely rare.

Regarding the causes of intradiaphragmatic abscesses, liver abscess after surgery for acute appendicitis,^4)^ complication of pneumatic dilatation for achalasia of the esophagus^1)^ and gallbladder surgery^3)^ have been reported. *Staphylococcus aureus* and *Escherichia coli* have been reported as causative bacteria of intradiaphragmatic abscesses.^1,3,4)^ Regarding localization, four of the five cases were on the right side, probably because due to associated hepatobiliary disease.^2–5)^ The present case was also on the right side. In this case, preoperative CT images and postoperative bacterial culture of pus suggested that it originated from inflammation spillover of residual gallstones after cholecystectomy. However, intraoperative findings showed mild adhesion between the diaphragm and the liver and no evidence of liver surface irregularity, suggesting that the inflammatory pseudotumor healed by the time the intradiaphragmatic abscess had formed. In addition, the bacterial culture of the pleural effusion was negative, and intraoperative findings suggested that the inflammation spillover from the intradiaphragmatic abscess likely extended into the thoracic cavity. Based on the above, this is the first case of intradiaphragmatic abscess formation in which we were able to capture the process of the formation of an intradiaphragmatic abscess due to the formation of a hepatic inflammatory pseudotumor caused by a residual gallstone.

A residual stone was found after cholecystectomy in this patient, but CT performed 6 months after surgery showed that the residual stone had disappeared, and an inflammatory pseudotumor was confirmed. At that time, the patient was asymptomatic, and blood tests were normal; therefore, follow-up was terminated at 6 months postoperatively. However, we believe that this hepatic inflammatory pseudotumor may have led to the formation of an intradiaphragmatic abscess, and continued follow-up may have prevented the formation of the intradiaphragmatic abscess and its subsequent surgical treatment. It is important to keep in mind that hepatic inflammatory pseudotumors can lead to the formation of intradiaphragmatic abscesses, as in this case.

It is reportedly very difficult to detect diaphragmatic lesions completely on CT,^6)^ and MRI has proven useful for dynamic evaluation of the diaphragm.^7)^ In our case, preoperative CT failed to completely identify the presence of an intradiaphragmatic abscess. Although preoperative MRI was performed in 2 previously reported cases,^4,5)^ one was misdiagnosed as a diaphragmatic tumor.^4)^ Thus, the diagnosis may be very difficult and often misinterpreted as a subdiaphragmatic abscess or loculated empyema.^1)^ In the present case, if MRI was performed preoperatively, an intradiaphragmatic abscess might have been recognized.

The principal treatment for an intradiaphragmatic abscess is drainage with the application of antibiotics.^1)^ In this case, we suspected pyothorax preoperatively; therefore, we used an abdominal as well as a thoracic approach. As a result, a combined abdominal and thoracic approach allowed us to make the correct diagnosis by locating an abscess within the diaphragm and performing appropriate treatment. Furthermore, although a combined abdominal and thoracic open approach was used for neonatal diaphragmatic abscess, as reported by Zouari et al.,^5)^ this is the first report of a surgery performed using only laparoscopic and thoracoscopic approaches.

In summary, the combined abdominal and thoracic approach is useful for diaphragmatic lesions, and it is important to prepare this approach as a preoperative surgical plan.

## CONCLUSIONS

When a subdiaphragmatic abscess or pyothorax is suspected, the presence of an intra-diaphragmatic abscess should always be considered, although this disease is extremely rare. Furthermore, if an intradiaphragmatic abscess is suspected, a combined abdominal and thoracic approach may be useful for making the correct diagnosis and appropriate treatment.

## ACKNOWLEDGMENTS

Not applicable.

## DECLARATIONS

### Funding

Not applicable.

### Authors’ contributions

Yuuki Matsui wrote the original draft and reviewed and edited the manuscript.

Koji Takami supervised, wrote, reviewed, and edited the manuscript.

All the authors have read and approved the final manuscript and have been involved in patient management.

### Availability of data and materials

The datasets supporting the conclusions of this article are included within the article.

### Ethics approval and consent to participate

All procedures followed were in accordance with the Helsinki Declaration. Informed consent to be included in this study was obtained from the patient.

### Consent for publication

Informed consent for publication of this case report was obtained from the patient.

### Competing interests

The authors declare that they have no competing interests.
